# Comparison of Non-Covalent and Covalent Interactions between Lactoferrin and Chlorogenic Acid

**DOI:** 10.3390/foods13081245

**Published:** 2024-04-19

**Authors:** Zekun Li, Majida Al-Wraikat, Changchun Hao, Yongfeng Liu

**Affiliations:** 1 College of Food Engineering and Nutritional Science, Shaanxi Normal University, Xi’an 710119, China; erkun218@163.com (Z.L.); majida@snnu.edu.cn (M.A.-W.); 2 College of Physics and Information Technology, Shaanxi Normal University, Xi’an 710119, China

**Keywords:** lactoferrin, non-covalent, covalency, laccase, spectroscopy

## Abstract

Adding polyphenols to improve the absorption of functional proteins has become a hot topic. Chlorogenic acid is a natural plant polyphenol with anti-inflammatory, antioxidant, and anticancer properties. Bovine lactoferrin is known for its immunomodulatory, anticancer, antibacterial, and iron-chelating properties. Therefore, the non-covalent binding of chlorogenic acid (CA) and bovine lactoferrin (BLF) with different concentrations under neutral conditions was studied. CA was grafted onto lactoferrin molecules by laccase catalysis, free radical grafting, and alkali treatment. The formation mechanism of non-covalent and covalent complexes of CA-BLF was analyzed by experimental test and theoretical prediction. Compared with the control BLF, the secondary structure of BLF in the non-covalent complex was rearranged and unfolded to provide more active sites, the tertiary structure of the covalent conjugate was changed, and the amino group of the protein participated in the covalent reaction. After adding CA, the covalent conjugates have better functional activity. These lactoferrin–polyphenol couplings can carry various bioactive compounds to create milk-based delivery systems for encapsulation.

## 1. Introduction

An iron-binding glycoprotein found in milk called bovine lactoferrin (BLF) has a high capacity to bind iron. Its molecular weight is around 80 kDa [1]. BLF is utilized extensively in the food and medical industries as a high-value dietary protein [2]. Studies have shown that BLF has antibacterial, anti-inflammatory, and anti-tumor properties in addition to its ability to regulate the immune system of mammals, lessen gastrointestinal discomfort, maintain iron balance, and promote the absorption of calcium, magnesium, and other nutrients [2,3,4,5] BLF controls cell growth and eliminates damaging free radicals [4]. The concentration of BLF in milk is 0.03–0.1 g L^−1^, and its structure is very similar to human lactoferrin, which has a concentration of about 1 g L^−1^ in human milk [6]. Due to its nutritional and health benefits, BLF has been added to many commercial foods and health supplements, such as infant formula, energy drinks, and certain dairy products [7]. Proteins can improve stability and delivery potential by cross-linking with chemical agents (such as glutaraldehyde) and enzymes (such as transglutaminase), but the high cost and toxicity of chemical agents limit applications [8]. Currently, most studies of food-grade protein–polyphenol conjugates focus on non-enzymatic methods, but little is known about the formation mechanism and functional properties of enzyme-formed conjugates [9].

Chlorogenic acid (CA) is a natural plant polyphenol with excellent antioxidant, anti-inflammatory, anti-obesity, and anti-tumor properties. It is a potential candidate for food formulations [10]. Studies have shown that polyphenols can strongly interact with proteins to improve protein stability and food quality [11]. Covalent and non-covalent interactions are the two main forms of polyphenol protein interaction. Non-covalent protein–polyphenol interactions are more common than covalent bonds, which include hydrogen bonds, hydrophobic interactions, and van der Waals gravitation [12]. Khan et al. investigated the interaction between sunset yellow dye and beta-lactoglobulin using various spectral and molecular modeling techniques. Thermodynamic results show that the interaction between sunset yellow dye and β-lactoglobulin is mainly hydrophobic [13]. Conjugates formed by covalent bonds are better suited for food applications because they have stronger, longer-lasting interactions and high stability [14]. Protein–polyphenol couplets can be covalently formed by non-enzymatic (alkaline reaction or radical grafting) or enzymatic (polyphenol oxidase, lucase, tyrosinase, etc.) methods. Polyphenols react with the amino group of the side chain of peptides under oxidation conditions, and molecular oxygen oxidizes the hydroxyl group on the polyphenol to quinone. The quinones interact irreversibly with the sulfhydryl and amino groups of proteins and further undergo condensation reactions [15]. Both enzymatic and non-enzymatic methods can be used to form protein–polyphenol conjugates. Jia et al. discovered that EGCG modification causes cross-linking in whey protein isolates, resulting in a more compact tertiary structure and better foaming and emulsifying properties [16]. Abd El-Maksoud et al. prepared multifunctional phenolic emulsifiers under different pH conditions using β-lactoglobulin (βLG) and caffeic acid as covalent coupling agents. They found that βLG-caffeic acid showed increased oxidation resistance, water solubility, and thermal stability compared to natural βLG and βLG/caffeic acid [17].

Therefore, studying the interaction between chlorogenic acid and lactoferrin could help optimize the formation of protein-based nanoparticles, thereby improving their delivery potential. The formation mechanism and functional properties of enzymatic and non-enzymatic conjugates and their differences from co-noncovalent complexes were investigated. The interaction between BLF and CA was studied using fluorescence spectrum, circular dichroism (CD), particle size and potential, SEM, and Fourier transform infrared spectroscopy (FTIR). The bonding between non-covalent molecules was predicted by molecular docking and molecular dynamics. The functional properties of CA-BLF complexes and conjugates were characterized by DPPH free radical scavenging activity, solubility, ANS fluorescence probe assay, differential scanning calorimetry, foamability and emulsification, and simulated digestion in vitro, and the formation mechanisms of different types of CA-BLF conjugates were compared.

## 2. Materials and Methods

### 2.1. Materials

Yuanye Biotechnology Co., Ltd. (Shanghai, China) was paid for bovine lactoferrin (BLF, HPLC ≥ 95%). Chlorogenic acid (CA, HPLC ≥ 98%) came from Chengdu Must Bio-technology Co., Ltd. (Chengdu, China) Kangwei Century Biotechnology (Beijing, China) Co., Ltd. and Beijing Solaibao Technology (Beijing, China) Co., Ltd. both sold BCA protein quantification kits and SDS-PAGE gel preparation kits. Coomas Bright Blue G-250 was purchased from Tianjin Comeiou Chemical Reagent (Tianjin, China) Co., Ltd. Rainbow 130 Broad-spectrum Protein marker (15–180 kDa) was purchased from Beijing Soleibao Technology Co., Ltd. It was decided to buy sodium dodecyl sulfate (SDS) from Tianjin Fuchen Chemical Reagent Factory (Tianjin, China) Co., Ltd. Protein was dissolved in phosphate-buffered saline (PBS, 0.1 mol L^−1^, pH 7.4). Analytical-grade reagents were also employed in the experiment, and ultra-pure water was used. In further studies, none of the samples underwent more purification.

### 2.2. Methods

#### 2.2.1. Reagent Configuration

The BLF protein solution was made into a 10 μM working solution with a PBS buffer. Chlorogenic acid was produced with a PBS buffer to create a working solution with a 500 μM concentration. The solution was stored in a brown glass tube and mixed in different proportions according to the needs of further experiments to obtain the CA-BLF physical complex [9,16,18].

As a reserve solution, distilled water was used to disperse BLF (1%, *w*/*v*, mass concentration 10 mg mL^−1^) and CA (0.2%, *w*/*v*, mass concentration 2 mg mL^−1^) powder, which was then fully hydrated for three hours. By using laccase catalytic oxidation, free radical grafting, and alkali treatment, respectively, the BLF-CA covalent complex was created [9].

(1) Oxidation catalyzed by laccase: After adjusting the pH of CA solutions to 4.5 ± 0.5, they were combined with BLF, in that order. The mixture was continually mixed for eight hours while exposed to oxygen at room temperature. After adding laccase (60 U g^−1^), the mixture was cultured for 16 h. “Lac-CA” was the sample’s recorded name.
(2) Free radical grafting: for three hours, use a magnetic stirrer to weigh, dissolve, and hydrate 60 mg BLF in 5.94 mL deionized water. The mixture was allowed to stand for two hours at room temperature and atmospheric pressure after adding 15 mg ascorbic acid and 0.06 mL of 5 mol L^−1^ hydrogen peroxide. After adding 0.021 mmol of CA, the reaction took place for 24 h at room temperature. “Rad-CA” was the sample’s record number.
(3) Alkali treatment: after using 0.1 M NaOH to bring the pH of the fully hydrated 1% (*w*/*v*) BLF and 0.2% (*w*/*v*) CA solutions to 9.0, respectively, the BLF and CA were combined in equal amounts while being continuously agitated (at 200 rpm). For a full day, the sample was continuously agitated and exposed to air at room temperature, which encouraged contact between the solution and oxygen. “Alk-CA” was the sample’s record number.

Using the physical complex of CA-BLF as the control, the preparation process involved simply combining equal amounts of BLF stock solution with CA. All covalent compounds and the physical complex were moved to a dialysis bag with a permeability of 7000 kDa. For 48 h, dialysis was performed at 4 °C, with water changes every 8 h to eliminate free polyphenols. After processing, all of the covalent complex samples were freeze-dried and kept for later use in a refrigerator; the remaining samples were kept at 4 °C.

#### 2.2.2. Determination of Total Phenol Content

A quantity of 0.5 mg of CA standard was accurately weighed, dissolved in deionized water, and filled to 5 mL in a volumetric flask. Deionized water was used to dilute the reserve solution and added to CA standard solutions with varying concentration gradients. Next, 2.25 mL of distilled water was added to 0.5 mL of these standard solutions, along with 0.25 mL of folin phenol reagent (2 N), and the mixture was left to incubate at room temperature for five minutes without exposure to light. After that, 7.5% (*w*/*v*) of Na_2_CO_3_ solution was added, and the mixture was left out of the light for two hours. Then the sample was dropped into a 96-well plate, and the absorbance at 760 nm was measured with an ELISA. The standard curve was established with the absorption value. After dispersing LF, the LF-CA physical complex, and the LF-CA covalent complex at the proper quantities into deionized water, the total phenol content of these samples was ascertained by applying the identical procedure outlined in the CA standard solution. The result was given in milligrams polyphenol equivalent per milliliter of sample [19]. The following Formula (1) was used to determine the grafting efficiency:

(1) $$Grafting\;efficiency\left(\%\right)=\left(C/C_{0}\right)\times 100$$

where *C* is the CA content in the physical complex or covalent complex, and *C*_0_ is the CA content in the pre-dialysis reactants.

#### 2.2.3. SDS-Polyacrylamide Gel Electrophoresis (SDS-PAGE)

SDS-PAGE was used to evaluate the digestion materials in 12% (*w*/*v*) acrylamide gels. Five distinct sample types were examined: physical, Lac-CA, Rad-CA, and Alk-CA.

The above samples were loaded into the ep tubes after being thoroughly mixed with a 4:1 volume-to-volume 5× SDS-PAGE loading buffer. They were then heated in a water bath at 100 °C for 3–5 min. It was then chilled for subsequent use in an ice bath. Each lane was loaded with aliquots of the following samples: marker, physical, Lac-CA, Rad-CA, and Alk-CA. Different settings were established at different voltages for separation and electrophoresis, respectively. The electrophoresis ceased when the indicator contacted the base. Subsequently, the gel was scraped, rinsed with deionized water, and stained with Coomassie Brilliant Blue G-250 in a shaker for two hours. The gel was taken out, washed with water that had been twice distilled, and then allowed to soak in a decolorizing solution all night. The decoloring solution was swapped out periodically.

#### 2.2.4. UV-Visible Spectrum

A Puxi T9CS double-beam UV–visible spectrophotometer was used to measure the UV absorption spectra of the samples. To produce nine distinct BLFs, dropwise addition of a 500 μM CA solution was made to the pure sample of protein after 4 mL of a 10 μM BLF protein sample solution had initially been added to a quartz cuvette (1:0, 1:1, 1:2, 1:3, 1:4, 1:5, 1:6, 1:8, and 1:10). For covalent complexes, a 1 mg mL^−1^ BLF sample solution was prepared using deionized water. The 240 to 400 nm spectral range was scanned.

#### 2.2.5. Fluorescence Spectrum

The fluorescence spectrum of a 4 mL sample of the BLF protein at a concentration of 10 μM was scanned using an RF-6000 fluorescence spectrophotometer (Island Tsu Company, Tsu, Japan) for non-covalent complexes. The protein was then added dropwise to the CA solution at a concentration of 500 μM to generate nine distinct BLFs: CA molar ratios (1:0, 1:1, 1:2, 1:3, 1:4, 1:5, 1:6, 1:8, and 1:10). At 280 nm, the excitation wavelength was set. The scanning range of 290–450 nm data was used to determine the emission wavelength. The excitation and emission light’s slit width were set to 5 nm: three distinct temperatures—290 K, 300 K, and 310 K—were used in the trials. 

A 1 mg mL^−1^ BLF sample solution was prepared using deionized water for covalent complexes. Fluorescence spectrum scanning was performed using a RF-6000 fluorescence spectrophotometer, in which the excitation wavelength was set to 295 nm, and the emission wavelength was set to 300–400 nm. The excitation and emission slit width was set to 5 nm.

#### 2.2.6. Circular Dichroism Experiment

A Characin CD spectrometer was used to gather CD spectral information from the protein solution. The circular dichroism experiment characterized the secondary structure of proteins in BLF, BLF-CA physical complex, and BLF-CA covalent complex. Far ultraviolet spectrum data were gathered by scanning the wavelength region between 190 nm and 260 nm. The data were processed using CDNN (version 2.1) software to create the circular chromatograms for each system and the contents of each protein secondary structure component.

#### 2.2.7. Fourier Transform Infrared Spectroscopy

The infrared spectra of BLF, BLF-CA non-covalent complex, and BLF-CA covalent complex were determined by Fourier transform infrared spectroscopy. The test sample was smeared onto Spectral-grade KBr tablets for analysis after being pulverized and compressed into tablets. The measurements, which had a resolution of 4 cm^−1^ and covered the range from 4000 to 400 cm^−1^, were made using a INVENNIO S Fourier infrared spectrometer (Bruker Daltonics, Bremen, Germany).

#### 2.2.8. Particle Size Distribution and Zeta Potential

The NanoBrook 90 PlusPALS laser particle sizer (Brookhaven Instruments, Holtsville, NY, USA) was used to examine the particle size and ζ-potential of the samples. Each measurement consisted of 15 scans taken over 120 s at a temperature of 298 K. Three measurements were taken in parallel for each sample, and the data were averaged. Technical abbreviations were explained upon first use.

#### 2.2.9. Scanning Electron Microscopy (SEM)

The microstructure of the samples was analyzed using field emission scanning electron microscopy (Nava NanoSEM 450, FEI, Brno, Czech Republic). To prevent static generation, the samples were coated with gold. Imaging of the samples was conducted at 10,000× magnification with an accelerating voltage of 15.0 kV.

#### 2.2.10. Molecular Docking and Simulation

**Molecular Docking Simulation.** Molecular docking simulation tests were carried out with the help of Auto Dock 4.2. In this experiment, the crystal structure of BLF (PDB ID: 1BIY) was downloaded from the Protein Data Bank (PDB) website [20]. After being loaded into pymol, it underwent a series of modifications to become an automatic docking-compatible system file, including removing water, adding hydrogen atoms, correcting erroneous amino acids, and adjusting charge. PubChem (https://pubchem.ncbi.nlm.nih.gov/, accessed on 6 November 2023) provided the CA structure, which was then translated to PDB format using Open Babel (PubChem CID: 1794427) [21]. The Lamarckian genetic algorithm (GA) was used for semi-flexible molecular docking, and we selected the grid box size to cover the complete protein at 126 × 126 × 114 Å. To find the best conformation after docking and further examine the microenvironment of the CA-BLF complex binding site, pymol was employed to investigate the predictions visually.

**Molecular Dynamics Simulation.** Gromacs2022.3 software was used for molecular dynamics simulation [22]. Gaussian 16 W was applied to hydrogenate small compounds and determine RESP potential, and AmberTools22 was utilized to apply the GAFF force field to tiny molecules. Potential information was added to the molecular dynamics system’s topology file. Atmosphere pressure of 1 bar and a static temperature of 298 K were used for the simulation. Amber99sb-ildn was utilized as the force field, water molecules were used as the solvent (Tip3p water model), and an appropriate amount of Na^+^ ions was added to balance out the system’s overall charge. With a coupling constant of 0.1 ps and a duration of 100 ps, the simulation system initially employed the steepest descent method to minimize energy. It then underwent both the isothermal isovolumic ensemble (NVT) equilibrium and the isothermal isobaric ensemble (NPT) equilibrium for 100,000 steps each. The simulation of free molecular dynamics was then completed. The operation took 10 ns to complete and had 5,000,000 steps with a step length of 2 fs. The software’s built-in tool was applied to examine the trajectory when the simulation was complete. Each amino acid trajectory’s root mean square variance (RMSD), root mean square fluctuation (RMSF), and protein rotation radius were merged with the free energy (MMPBSA), free energy topography, and other data [23]. 

#### 2.2.11. DPPH Radical Scavenging Activity

A 1.2 × 10^−4^ mol L^−1^ DPPH alcohol solution was prepared with ethanol, about 2 mL of the diluted sample (0.5 mg/mL) was mixed with 2 mL of DPPH ethanol solution, and then the mixture was stored in the dark. After 60 min, the residual DPPH concentration was determined. After finishing, the sample was dropped into the 96-well plate, and the absorbance at 517 nm was measured using an enzyme label [16,24]. Results were expressed as radical scavenging activity calculated as follows:

(2) $$DPPH\;free\;radical\;scavenging\;activity\;\left(\%\right)=\frac{A_{0}-\left(A_{2}-A_{1}\right)}{A_{0}}\times 100$$

*A*_0_ represents the absorbance when DPPH is added to the sample with distilled water. Additionally, A_1_ represents the absorbance when DPPH is added to the sample with ethanol, and A_2_ represents the absorbance of the sample.

#### 2.2.12. Surface Hydrophobicity

Using a bionic fluorescent probe,-8-sulfonic acid (ANS), was added to 3 mL of the sample in a dark reaction for 15 min. Fluorescence intensity was measured by monitoring the excitation wavelength of 375 nm and the emission wavelength range of 390–650 nm.

#### 2.2.13. Determination of Solubility

The complexes and concatenates (pH 7.0) were centrifuged at room temperature for 20 min at 10,000× *g*. The protein content of the supernatants and raw samples was determined using the bicinchoninic acid (BCA) assay.

(3) $$Solubility\;\left(\%\right)=\frac{Soluble\;protein\;content}{Total\;protein\;concentration}\times 100$$

#### 2.2.14. Measurement of Thermal Stability

Calorimetric analysis was performed using the DSC Professional Thermal Analysis system (Mettler Toledo, Columbus, OH, USA). In the standard procedure, about 1 mg of the sample was placed in an aluminum pan, sealed tightly with a perforated aluminum lid, heated from 30 °C to 180 °C at a constant rate of 10 °C min^−1^, and dry nitrogen was constantly removed at a rate of 30 mL min^−1^. An empty aluminum pan was used as a reference [19]. The denaturation peak temperature of each thermal curve was calculated by using general analysis software.

#### 2.2.15. Determination of Foaming Properties and Emulsifying Properties

Transfer 15 mL of non-covalent complexes and covalent splices into 100 mL plastic measuring cylinders. The mixture homogenized using a high-speed homogenizer at 10,000 rpm for 1 min at room temperature. The total volume was measured immediately after whipping and after, the specimens were allowed to stand for 30 min. Foam expansion (*FE*) and foam stability (*FS*) were calculated using the following equation:

(4) $$FE\left(\%\right)=(V/V_{0})\times 100$$

(5) $${FS\left(\%\right)=(V}_{30}/V_{0})\times 100$$

where *V* is the total volume after high-speed mixing; *V*_0_ is the original volume before high-speed mixing, and *V*_30_ is the total volume after 30 min of resting.

A mixture of 5 mL of peanut oil and 15 mL of each sample was homogenized in a plastic cylinder using a high-speed homogenizer at 20,000 rpm for 1 min. Subsequently, 50 μL of each mixture was withdrawn from the bottom of the cylinder at 0 and 10 min, respectively, and added to 1 mL of 0.1% (*w*/*v*) sodium dodecyl sulfate (SDS) solution. The mixture was thoroughly mixed, and the absorbance was measured at 500 nm using an enzyme meter. The emulsification activity index (*EAI*) and emulsification stability index (*ESI*) were calculated using the following formulas:

(6) $$EAI\;\left(m^{2}/g\right)=\left(2\times 2.303\times A_{0}\times DF\right)/\left(L\times \Phi \times c\right)$$

(7) $$ESI\;(min)=(A_{0}\times 10)/\left(A_{0}-A_{10}\right)$$

where *A*_0_ and *A*_10_ denote the absorbance of dispersion obtained at 0 and 10 min, respectively. *DF* denotes the dilution factor; *L* is the path length of the test tube (m); *Φ* is the oil volume fraction; and *c* is the protein concentration (g/mL).

#### 2.2.16. Measurement of Biological Accessibility

An in vitro digestion system with oral, gastric, and intestinal phases was used to track the digestive patterns of BLF and the CAF-BLF complex. The activity of digestive enzymes and the concentration of bile salts at each stage of digestion were measured experimentally or by recommended standardized measurements [25,26]. The INFOGEST method was used to simulate the digestive process in vitro [25]. At the end of the in vitro simulated digestion, the final digest was sampled and the bioaccessibility of lactoferrin was calculated according to the following equation:

(8) $$Bioaccessibility\;(\%)=\left(C_{digesta}/C_{initial}\right)\times 100$$

where *C*_digesta_ is the concentration of BLF in the intestine after digestion in vitro; *C*_initial_ is the initial concentration of BLF.

#### 2.2.17. Statistical Analysis

The means ± standard deviation were computed using the triplicate analysis findings. SPSS Statistics (Version 26) software was used to statistically analyze all the data, and Duncan’s multiple range test was used to determine significant differences at the level of *p* < 0.05. 

## 3. Results and Discussion

### 3.1. Formation of Covalent and Non-Covalent Complexes

#### 3.1.1. Total Phenol Content

To characterize the amount of CA binding to BLF, the complex was dialyzed to remove the unreacted CA, and then the total phenol content of the sample was detected. The equivalent of CA was calculated by the linear fitting equation y = 9.935x + 0.1077 (*R*^2^ = 0.9989) (y is the absorbance at 760 nm and x is the content of CA, in mg/mL, respectively). The CA grafting efficiency of the CA-BLF non-covalent complex and the CA-BLF covalent complex prepared by three methods is shown in Table 1. The grafting efficiency of CA-BLF was 34.70%, while that of laccase-catalyzed, free radical-grafted, and alkali-treated covalent complexes were 51.39%, 16.82%, and 82.40% respectively. Since non-covalent interactions between proteins and polyphenols (mainly hydrogen bonding and hydrophobic–hydrophobic interactions) are generally reversible and weaker than covalent interactions, the grafting rate of non-covalent complexes is lower [11]. The grafting rate of the complex treated by free radical grafting is much lower than that of the non-covalent complex, indicating that the binding efficiency of BLF and CA in the complex treated by free radical grafting is very low, which is much lower than that of the other three methods. The grafting rate of alkaline conjugates was the highest, which was significantly higher than that of the conjugates catalyzed by free radicals and laccase (*p* < 0.05), indicating that alkali treatment promoted the covalent binding of CA and BLF more effectively.

#### 3.1.2. SDS-PAGE

**Figure 1:**
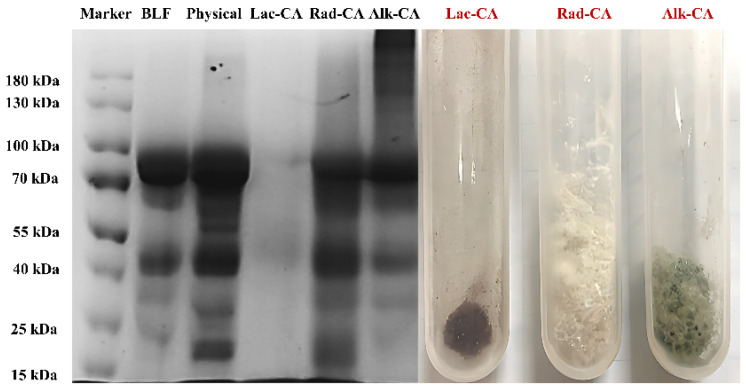
SDS-PAGE Diagram of BLF, BLF-CA physical complex, and BLF-CA covalent complex.

As SDS and mercaptoethanol are known to cleave non-covalent and disulfide bonds, covalent conjugate formation is determined by electrophoresis (SDS-PAGE) [9]. SDS-PAGE analysis (Figure 1) showed that BLF had a strong band around 80 kDa, which was consistent with the known molecular weight of the protein. In contrast, the location of the protein band in the BLF-CA complex was not significantly different from that of BLF, indicating that the non-covalent bond in the physical complex is broken [27]. The band analysis of the covalent complex formed by BLF and CA showed that the bands of Rad-CA and Alk-CA contained a main band whose molecular weight was slightly higher than that of pure protein. Alk-CA also contained some dispersion bands with much higher molecular weight than pure proteins, while CA had relatively low molecular weight, suggesting that alkali treatment can enable CA and lactoferrin to form higher molecular weight couplers. At the same time, the main band of Lac-CA was at 90 and 40–60 kDa, indicating that laccase may have destroyed most of the structure of lactoferrin, causing it to be decomposed into small molecular weight peptide segments. However, there were still a few intact Lac-CA covalent complexes. This may be the reason that the sample morphology of Lac-CA is different from that of other groups. As shown in the freeze-dried sample diagram on the left of Figure 1, the Lac-CA sample was crystalline, and Rad-CA and Alk-CA samples were wadded. The crystalline powder made Lac-CA difficult to redissolve in water after freeze-drying, and there was a phenomenon that the sample accumulated in the sample cell without sinking during electrophoresis.

### 3.2. UV–Visible Spectrum

An inexpensive, straightforward, adaptable, and nondestructive technique for analyzing organic materials and some inorganic content is ultraviolet–visible absorption spectroscopy [28]. It investigates how proteins change structurally and how their complexes form. In UV–Vis spectroscopy, proteins and polyphenols can be absorbed. The structure of the protein skeleton is reflected at 200 nm. It is an aromatic amino acid at 280 nm. Electron transitions between π molecular orbitals are typically responsible for their UV–visible absorption spectra. In the UV–visible region, organic compounds obey transition rules of σ–σ*, n–σ*, n–π*, and π–π*. In the ultraviolet area, the σ–σ* and n–σ* transitions are typically below 200 nm and are not easily observable. Absorption spectra for organic compounds are principally based on n–π* and π–π* transitions owing to the presence of unsaturated functional groups, which excite the relatively more easily stimulated n and π electrons [29]. Hence, UV–Vis spectroscopy investigations investigated the interaction mechanism between CA and BLF.

**Figure 2:**
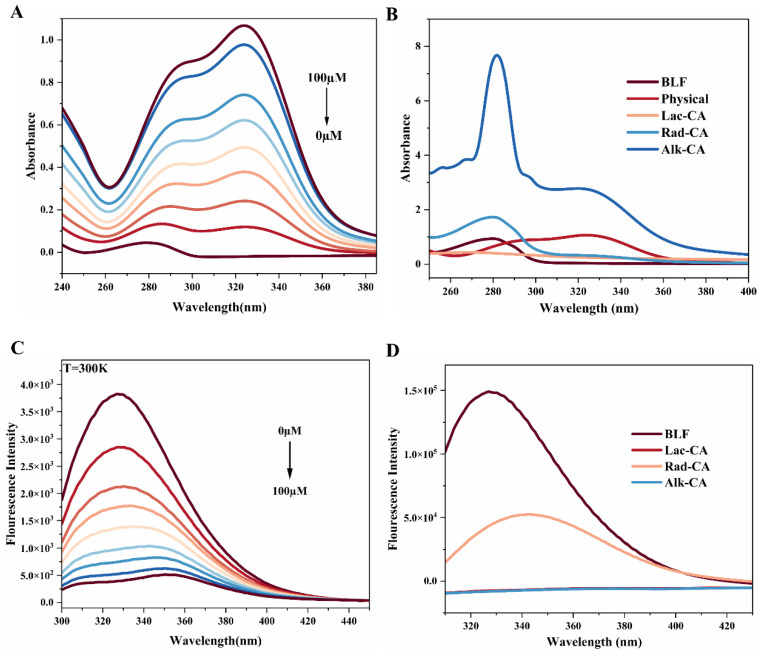
(**A**) UV–Vis spectra of non-covalent complexes formed by combining CA and BLF at different concentrations. (**B**) UV–Vis spectra of CA-BLF covalent complexes. (**C**) Fluorescence quenching spectra of CA-BLF physical complexes at 300 K. (**D**) Fluorescence spectra of CA-BLF covalent complexes.

The UV–Vis spectra of the physical complex of BLF at different chlorogenic acid doses are shown in Figure 2A. At first, the peak intensity of CA absorption in the wavelength range of the protein UV spectrum could be largely ignored. Secondly, as the CA content increased, there was a significant increase in the UV absorption intensity of BLF amino acid residues at 280 nm. The UV absorption spectrum of the CA-BLF composite system showed a very obvious redshift, and the maximum absorption peak moved from 280 nm to 325 nm. The redshift of this absorption peak may be due to the interaction of CA with hydrophobic amino acid residues of BLF molecules to form hydrophobic cavities. The regular change in UV absorption spectra after CA addition further indicated that CA and BLF could interact to form physical complexes, but further studies are needed to confirm the interaction mechanism of the complex system. Next, we used variable temperature fluorescence experiments to calculate further.

The UV–Vis spectrum of the CA-BLF covalent complex is shown in Figure 2B. Except for the laccase-catalyzed CA-BLF, the other groups resulted in a significant increase in the UV absorption intensity of BLF amino acid residues at 280 nm, which was due to the conjugated system of benzene rings on aromatic amino acids and the gradual increase in polyphenols. The attenuation of UV absorption of CA-BLF catalyzed by laccase may be due to increased steric hindrance or n–π* transition. The absorbance and wavelength of the CA-BLF complexes’ characteristic peaks increased upon treatment with alkali. This finding pointed towards increased benzene rings combined, a stronger conjugated system, and the possibility of π–π* transition occurring.

### 3.3. Fluorescence Spectrum

#### 3.3.1. Fluorescence Quenching Type of CA-BLF Physical Complex

The fluorescence emission of a protein solution containing these fluorophores is typically decreased when ligands are added due to the fluorescence quenching of small molecules. However, the inner-filter effect (IFE), which occurs when the ligand appropriately absorbs light in the excitation and emission wavelength range, has a significant impact on the UV–Vis absorption capabilities of the ligand and the fluorescence attenuation of the protein [30]. The fluorescence intensity was adjusted using the following equation to remove the inner-filter effects:

(9) $$F_{cor}=F_{obs}\times 10^{-\frac{A_{ex}\times d_{ex}}{2}-\frac{A_{em}\times d_{em}}{2}}$$

*A_ex_* and *A_em_* are the quencher and lactoferrin’s absorption intensities at excitation and emission wavelengths, and *F_cor_* and *F_obs_* are the fluorescence intensities observed and corrected, respectively.

A variety of quenching methods can explain the behavior, but the two that are most frequently used are dynamic quenching and static quenching. Dynamic quenching is the reduction in fluorescence intensity without changing the protein structure caused by the interaction of quenching small molecules with fluorescent excited state proteins. The formation of a non-emissive ground state complex between fluorescent molecules and ground state quenching molecules leads to static quenching of the fluorescence quenching process, which diminishes fluorescence and induces structural shifts in proteins [31]. Since greater temperatures might lead to bigger diffusion coefficients, the bimolecular quenching constants will rise in the dynamic quenching process as the temperature rises. In contrast, because complexes become less stable as temperature rises, the static quenching constants are anticipated to decrease [26]. Dynamic quenching and static quenching can be identified based on these traits. To further explore, we used the Stern–Volmer Equation (10) to process experimental data:

(10) $$\frac{F_{0}}{F}=1+K_{SV}[Q]=1+K_{q}\tau_{0}[Q]$$

where *F*_0_ and *F* are lactoferrin’s strengths with and without a quenching agent, respectively. The quencher concentration is *Q*. The Stern–Volmer quenching constant is *K_sv_*. The quenching rate constant is *K_q_.* It is lactoferrin’s typical half-life, which is 10 ns when the quencher is not present. By performing a linear regression on the *F*_0_/*F* and *Q* pictures, the Stern–Volmer equation can be used to calculate the value of *K_sv_*.

Figure 2C shows the effect of chlorogenic acid on lactoferrin at 300 K. The interaction between chlorogenic acid and lactoferrin caused the fluorescence intensity of lactoferrin to decrease significantly with the increase in chlorogenic acid concentration.

There was a good linear relationship between *F*/*F*_0_ and chlorogenic acid concentration. The quenching process rate constant *K_q_* was calculated at 290 K, 300 K, and 310 K temperatures (see Table 2). It could be seen from the experimental data that the value of *K_q_* was much higher than the dynamic quenching constant of 2.0 × 10^10^ L mol^−1^ s^−1^ for the maximum diffusion collision. Therefore, the technique of chlorogenic acid quenching lactoferrin fluorescence might be static quenching rather than dynamic quenching.

#### 3.3.2. Binding Constant and Binding Site Analysis of CA-BLF Physical Complex

Equation (11) can be used to explain static quenching. The slope and intercept of *log*(*F*_0_ − *F*)/*F* and *log*[*Q*] in the double-logarithm equation can be used to calculate the binding constant *K_A_* and the binding site n for static quenching:

(11) $$log\frac{F_{0}-F}{F}=nlogK_{A}-nlog\left(\frac{1}{\left[Q\right]-\frac{\left(F_{0}-F\right)\left[P\right]}{F_{0}}}\right)$$

where [*P*] is the concentration of BLF, [*K_A_*] is the binding constant, *n* is the number of binding sites, and *F*_0_ and *F* are the strengths of lactoferrin with or without a quenching agent. The quencher concentration is *Q*.

Figure 2C shows the *log*((*F*_0_ − *F*)/*F*) and *log*(1/([*Q*] *−* [*P*] (*F_0_ − F*)/*F*_0_)) relationships for quenching at different temperatures, where *K_A_* and *n* can be determined by the Y-intercept and slope, as shown in Table 2. Since *K_A_* decreased with the increase in temperature, it was further indicated that the quenching between CA and BLF was static quenching. In addition, drugs that were essentially bound to proteins typically have *K_A_* values between 10^5^ and 10^7^ L mol^−1^, while drugs that were moderately or weakly bound to proteins have *K_A_* values between 10^2^ and 10^4^ L mol^−1^ [32]. As a result, the binding force between CA and BLF was medium to moderate in the body.

#### 3.3.3. Thermodynamic Analysis and Interaction Force of CA-BLF Physical Complex

Hydrogen bonds, electrostatic forces, van der Waals forces, and hydrophobic interactions are among the mechanisms through which tiny molecules and proteins interact. One way to assess this interaction is to examine the change in thermodynamic characteristics. The calculation equation was as follows:

(12) $$\log K_{A}=-\frac{\Delta H}{2.303RT}+\frac{\Delta S}{2.303R}$$

(13) ΔG=ΔH−TΔS

where *K_A_* is the connection constant in Equation (13), *T* is the thermodynamic temperature, *R* is the gas’s stability, Δ*H* and Δ*S* are the changes in enthalpy and the amount of entropy shift, respectively, and Δ*G* is the Gibbs free energy. 

The values of Δ*H* and Δ*S*, after a linear fit of the logarithm *K_A_* to 1/*T*, were listed in Table 2. Using Equation (12), the values of Δ*G* were determined and listed in Table 2. Δ*H* < 0 and Δ*S* > 0 indicated that the electrostatic interaction seems to be the main internal force between chlorogenic acid and lactoferrin, and the Δ*H* and Δ*S* values of CA and BLF were −1.35 kJ mol^−1^ and −85.79 kJ mol^−1^, respectively [33]. Δ*H* < 0 indicated that the reaction was exothermic, and Δ*G* < 0 indicated that the reaction between chlorogenic acid and lactoferrin was spontaneous.

In summary, the electrostatic interaction appears to be the main internal force between the chlorogenic acid and lactoferrin physical complex and the reaction is spontaneous exothermic.

#### 3.3.4. Intrinsic Fluorescence Emission Spectrum of CA-BLF Covalent Complexes

The intrinsic fluorescence emission spectrum of proteins is usually dominated by tryptophan (Trp) and provides sensitive detection of the tertiary conformation of the relevant protein [16]. Environmental changes in these tryptophan residues lead to changes in the fluorescence spectra, so this method can provide information about structural changes. BLF is reported to have 13 tryptophan residues in its natural state [9]. The intrinsic tryptophan fluorescence determination was performed to understand better the conformational changes caused by the reaction of BLF and CA. The effects of laccase catalysis, radical grafting, and alkaline treatment on the fluorescence emission spectra of lactoferrin are shown in Figure 2D. The maximum fluorescence emission spectrum of lactoferrin was changed from about 330 nm to about 340 nm by radical grafting, and the fluorescence intensity was significantly reduced and redshifted. This suggested that the globular protein partially unfolded due to perturbations in the protein’s tertiary structure, resulting in some tryptophan residue being exposed to a more hydrophilic environment after modification. Therefore, the fluorescence results confirmed that chlorogenic acid was covalently coupled to BLF by the hydrogen peroxide–ascorbic acid pair as a radical initiating system. This was consistent with Kristo et al.’s study, which showed a redshift of 9 nm after radical grafting of catechins [34]. Lac-CA and Alk-CA were completely quenched, possibly because the Trp residues in the modified BLF were less exposed and were buried deeper in the protein core [34]. It may also be the interaction of the aromatic rings of phenolic compounds with the aromatic residues of proteins such as tyrosine and tryptophan [35].

### 3.4. Circular Dichroism Experiment

The alteration in the secondary structure can be seen in the far-ultraviolet band (200–250 nm) [36]. A common technique for determining the secondary structure of proteins is circular dichroism.

**Figure 3:**
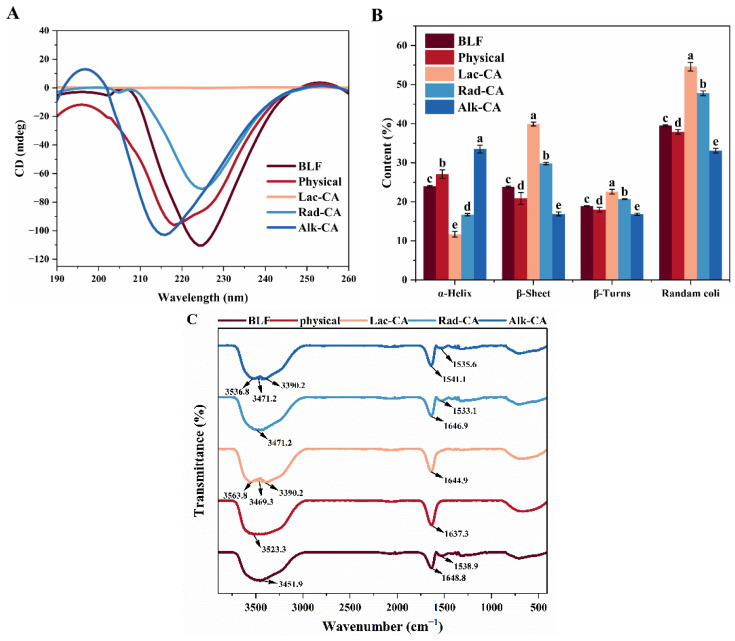
(**A**) CD spectra for BLF, BLFCA, and BLFCA conjugates. (**B**) Secondary structure of BLF, BLFCA, and BLFCA conjugates. (**C**) FTIR spectra for BLF, BLFCA, and BLFCA conjugates. Different lowercase letters in the figure indicate significant differences (*p* < 0.05).

The secondary structure content of the BLF-CA complex and conjugate was studied by far-ultraviolet CD spectroscopy. As shown in Figure 3A, a wide negative band of 210–240 nm was observed in the far-ultraviolet CD spectrum of BLF. Both covalent and non-covalent reactions between CA and BLF resulted in a decrease in the ellipticity of this negative band, indicating that the secondary conformation of BLF had changed. However, the far-ultraviolet spectrum’s peak of the conjugated compounds shifted slightly, and the negative band’s ellipticity decreased notably within the 210–220 nm wavelength range. This shift indicated that covalent interaction altered the protein’s secondary structure, while non-covalent interaction had a minor impact. It was worth noting that the Lac-CA image was almost a straight line, possibly due to over-catalysis by laccase and being combined with the electrophoretic map. It showed that BLF was broken down into small peptides, making the CD curve less obvious. The secondary structure composition of the sample was evaluated with CDNN 5.1. Figure 3B shows the components of α-helix, β-sheet, β-turn, and disordered structures in BLF, BLF-CA complexes, and BLF-CA couplings. The α-helix structure of BLF accounted for 24.0%, the β-sheet structure 23.8%, the β-turns structure 18.9%, and the random coiled structure 39.5%. In this study, Lac-CA and Rad-CA reduced the α-helix and increased the rest of the structure, in contrast to Alk-CA, suggesting that changes in the secondary structure of the protein may depend on the preparation method used to create the conjugate. The random coil content of Rad-CA and Lac-CA was increased, suggesting that laccase and hydrogen peroxide/ascorbic acid pair-induced covalent binding of CA to BLF reduced the proportion of ordered regions in the protein structure [9]. Consistent with the study of Wu et al., the α-helix content decreased slightly after the radical grafting coupling of β-lactoglobulin and CA. In contrast, the content of the unfolded structure increased [37] Lac-CA and Rad-CA produced more β-sheet structures, indicating that the treatment of laccase and free radical grafting made the structure of BLF more compact [4].

### 3.5. Fourier Transform Infrared Spectroscopy

FTIR was used to further study the structural changes in BLF combined with CA. FTIR can provide comprehensive information regarding chemical composition [38]. The advantages of FTIR over circular dichroism spectroscopy include the ability to analyze protein structure in solids, crystals, and aqueous solutions, and the effectiveness of each wave number’s absorbance. Three important characteristic absorption bands are included in the FTIR spectrum: 1700–1600 cm^−1^ (amide I band) represents the tensile vibration of C=O, 1600–1500 cm^−1^ (amide II band) represents the bending vibration of N-H and C-H, and 1300 cm^−1^–1260 cm^−1^ represents the vibration of C-O and C-O-C [20]. The FTIR spectra of the BLF-CA complex and conjugate were similar to those of BLF, confirming that they both contained this protein. The spectra of the unmodified BLF associated with the secondary structure of the protein show major bands of 1648.8 cm^−1^ (amide I) and 1538.9 cm^−1^ (amide II) (Figure 3C). Compared with the control protein, the amido I and amido II bands of BLF shifted after CA addition, indicating a change in the secondary structure of BLF in the complex and conjugate. The addition of chlorogenic acid increased the peak value of lactoferrin, and C=O was stretched. BLF also had 3451.9 cm^−1^ (an amide A band, representing the stretching vibration of the NH group). Compared with BLF, the amide A band of the BLF−CA complex and conjugate was slightly shifted, indicating that the -NH_2_ of the protein in the conjugate was involved in the covalent reaction. These FTIR results were consistent with fluorescence and CD spectra.

### 3.6. Particle Size Distribution and Zeta Potential

**Figure 4:**
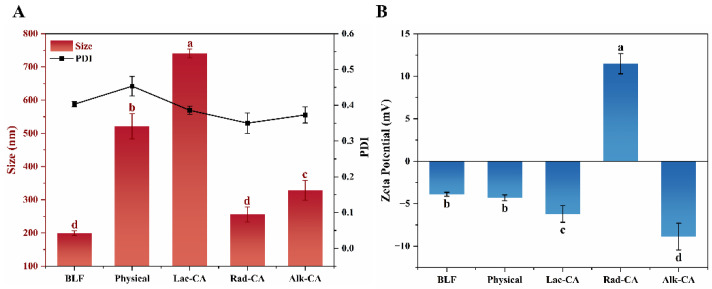
(**A**) Particle size and PDI; (**B**) ζ-potential of CA-BLF non-covalent and covalent complexes. Different lowercase letters in the figure indicate significant differences (*p* < 0.05).

Figure 4A displays the particle size and PDI of CA-BLF complexes and splices. Both non-covalent and covalent complexes had significantly larger particle sizes than BLF (*p* < 0.05). The increase in particle size of the non-covalent complexes suggests that CA and BLF may form larger aggregates through hydrophobic interactions, leading to an increase in particle size [39]. Furthermore, in the presence of laccase, H_2_O_2_, or under alkaline conditions, CA can cause cross-linking of BLF, resulting in the formation of dimers. The sulfhydryl or amino side chains of the peptides may also interact with the dimers to establish covalent C-N or C-S interactions with the phenolic ring, leading to an increase in particle size [40]. As can be seen from the PDI, the particle size of the samples with covalent interactions is more homogeneous than under non-covalent interactions. This indicates that covalent interactions are irreversible while non-covalent interactions are reversible. This corresponds to the results for polyphenol binding equivalents. The Rad-CA covalent complex had the smallest PDI, as low as 0.35, indicating the best homogeneity of the environmental system after free radical grafting.

The zeta potential is used to measure the charge on the surface of a dissolved particle. Proteins become amphoteric ally ionized when in solution. The isoelectric point of BLF is approximately 8.0 [41]. The absolute values of the potentials of the complexes and splices are greater than that of BLF, as shown in Figure 4B. The surface potentials of all groups are negative, except for Rad-CA. When more OH^−^ is present in the solution, the pH of Alk-CA becomes greater than its isoelectric point, resulting in a negative charge on the protein. The high negative charge of the protein itself, as well as its binding to many negatively charged CA molecules, may contribute to the physical and Lac-CA types. The pH of the solution is reduced by the presence of ascorbic acid in Rad-CA, and the low content of bound polyphenols causes the solution surface potential to be positive.

### 3.7. SEM

**Figure 5:**
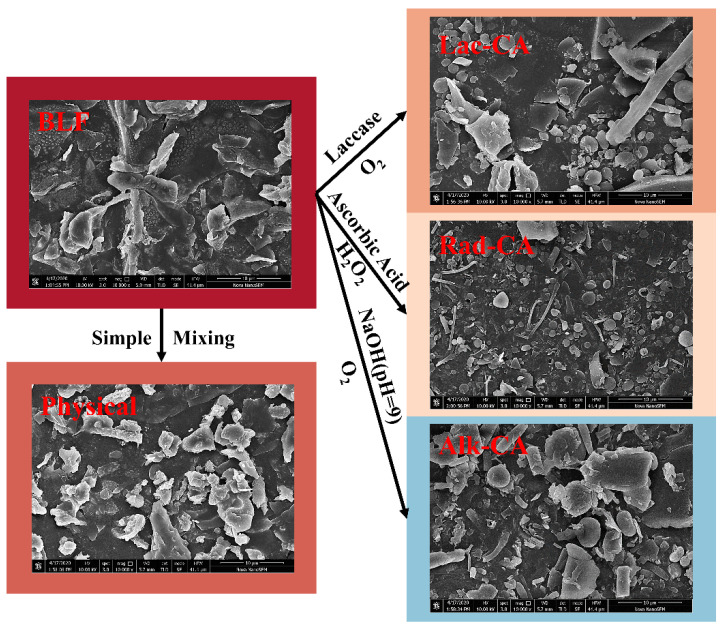
SEM images of CA-BLF non-covalent and covalent complexes (scale = 10 μm).

SEM was used to observe the microstructure of the sample. Figure 5 shows that adding CA resulted in a smoother and more spherical appearance. The covalent complexes formed smaller particles with smoother surfaces compared to the non-covalent complexes of lactoferrin, indicating that the lactoferrin molecules’ structure was altered by the polyphenols. Lac-CA and Rad-CA exhibit more spherical structures with a uniform distribution and compact structure. This indicates that the self-assembly of lactoferrin molecules is most affected by laccase catalysis and free radical grafting, leading to aggregation between CA and BLF.

### 3.8. Binding Mechanisms of Covalent and Non-Covalent Compounds

#### 3.8.1. Binding Mechanisms of Covalent Compounds

An enzymatic free radical initiation reaction and a subsequent non-enzymatic binding reaction are involved in the laccase-catalyzed oxidation reaction. Free radicals in CA can be generated by laccase and then react with proteins to produce protein–polyphenol covalent complexes. The redox pair of ascorbic acid and hydrogen peroxide initiates the free radical grafting process. Hydroxyl radicals are created when ascorbic acid and hydrogen peroxide combine [16,18]. Subsequently, these radicals target the amino groups in the protein side chain and the hydrogen atoms in the sulfhydryl, creating a reactive intermediate (a protein radical). A covalent link is created between the protein and polyphenol as a result of this intermediate’s reaction with the aromatic ring of CA. The aromatic ring of CA has o- and p-hydroxyl groups that are highly reactive with free radicals. The mechanism of the reaction is comparable to alkali therapy. The mechanism of the treatment process entails causing the hydroxyl group in phenol to deprotonate and generate a quinone. After that, this quinone combines with phenolic substances to create dimers or covalent connections with the protein’s nucleophilic groups [9].

#### 3.8.2. Binding Mechanisms of Non-Covalent Compounds (Molecular Docking and Simulation)

**Figure 6:**
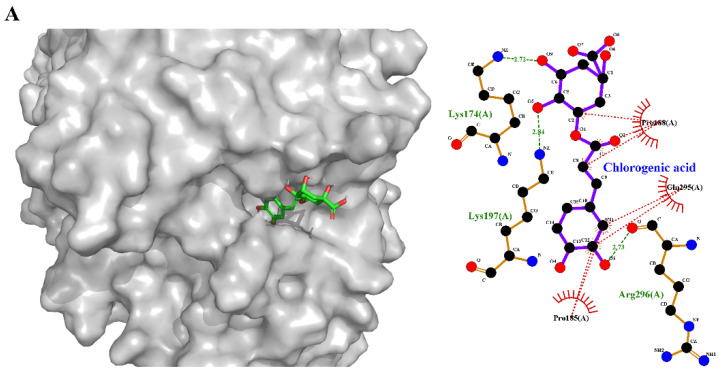

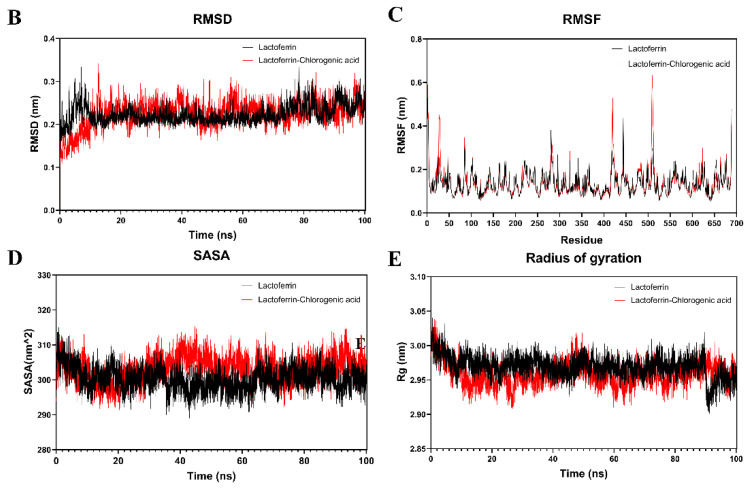
(**A**) Molecular docking of CA-BLF. (**B**) Root mean square deviation (RMSD) of chlorogenic acid molecule interaction with lactoferrin. (**C**) Root mean square fluctuation (RMSF) of chlorogenic acid molecule interaction with lactoferrin. (**D**) SASA of chlorogenic acid molecule interaction with lactoferrin. (**E**) Cyclotron radius of chlorogenic acid molecule interaction with lactoferrin.

Molecular simulations can accurately predict binding locations and interactions between relevant molecules [42]. To assess the binding energy and contact forces of protein–ligand non-covalent complexes, determine the presumed binding site of the ligand, and study the microenvironment of the binding site, we used molecular docking techniques. As shown in Figure 6A, CA and BLF used the simulated docking model with the minimum binding energy (this way, the combination stability was the best). The CA and BLF active sites were closely related to it. Docking studies showed that lactoferrin residues Lys174, Lys197, Pro185, Arg296, Gln295, and Pro188 were involved in the interaction with CA and bound to Lys174, Lys197, and Arg296 through hydrogen bonding. The remaining three residues, Pro185, Gln295, and Pro188, are bound to CA through hydrophobicity.

On the other hand, by choosing the appropriate force field, solvent, pH, ion presence, and other experimental parameters [43]. To evaluate the stability and interaction mechanism of CA molecules binding to BLF, a 100,000 ps molecular dynamics simulation was performed. The root mean square deviation (RMSD) between the main atom and the initial structure of the protein was analyzed to detect the trajectory stability of the CA molecule after binding to the protein. As shown in Figure 6B, after the 20,000 ps simulation, RMSD values of the CA molecule and protein tended to be stable and low, which was close to the stability of the protein molecule, indicating that the CA molecule had high binding stability. The results were consistent with the stoichiometric results of thermodynamic tests [22,23].

RMSF measures the average of changes in atomic positions over time and can describe the flexibility and movement of protein amino acids throughout the simulation. As shown in Figure 6C, the RMSF value of BLF decreased slightly after binding to CA, indicating that the protein structure changed to some extent and had higher stability. This was consistent with the experimental results of the circular dichroism experiment [22,23].

Solvent-accessible surface area (SASA) refers to the surface area of biomolecules exposed to the solvent. In the simulation, the scale of the probe radius was less than 1.4 Å [22,23]. As shown in Figure 6D, the changes in SASA indicated that the microenvironment and surface hydrophobicity of the CA-BLF complex changed during the molecular simulation process, and finally reached stability.

The cyclotron radius may describe the compactness of the protein structure and alterations in the looseness of the protein–peptide chain during simulations. Molecular dynamics simulations employ Rg to assess the firmness and inflexibility of the protein backbone, which may further reveal protein stability at varying temperatures [22,23]. As can be seen from Figure 6E, after the 60,000 ps simulation, the structural cyclotron radius of the CA-BLF complex tended to be stable, indicating that the complex had good structural tightness and stability.

MM/PBSA was used to calculate binding energy values and interactions between ligands and proteins. The binding energy (ΔG_MMGBSA_) of lactoferrin–chlorogenic acid was −65.77 kJ mol^−1^, indicating that lactoferrin was automatically bound to CA. The electrostatic force between lactoferrin and chlorogenic acid was −111.17 kJ mol^−1^ and the van der Waals force was −99.16 kJ mol^−1^. In addition, there were three hydrogen bonds between lactoferrin and chlorogenic acid, and the dissociation constant was 2.91 × 10^−12^ M. The results showed that the electrostatic effect of lactoferrin and chlorogenic acid was slightly stronger than the van der Waals force. The binding of CA to lactoferrin, hydrogen bond, and electrostatic effect were very important. Thus, the results confirm the discovery of quenching fluorescence (as shown by both molecular dynamics simulations and thermodynamic experiments), and the electrostatic interaction was the main intrinsic binding force between chlorogenic acid and lactoferrin.

### 3.9. DPPH Radical Scavenging Activity

One of the most crucial characteristics of protein–polyphenol conjugates is antioxidant activity. Protein polyphenol couplings have been found to have higher antioxidant activity than the native proteins in numerous investigations [44]. Therefore, the antioxidant properties of BLF, BLF-CA covalent, and non-covalent complexes were determined by the DPPH radical scavenging method.

**Figure 7:**
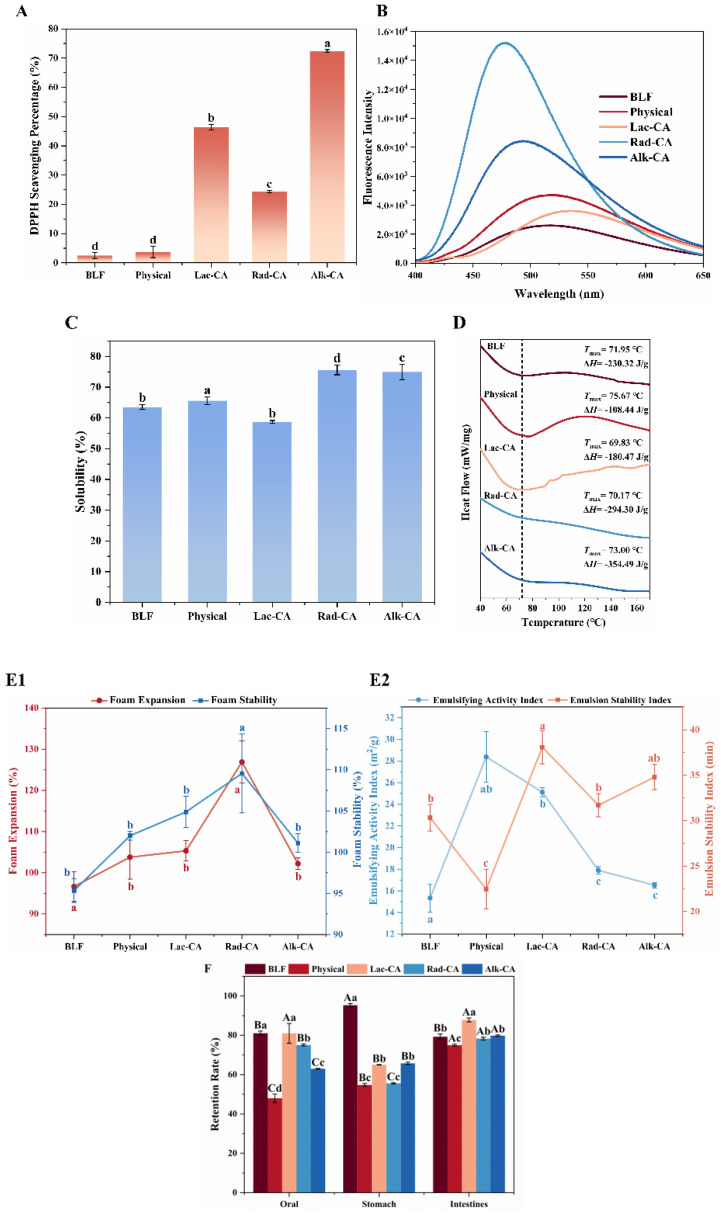
(**A**) Scavenging activity of BLF, BLF-CA physical complexes, and BLF-CA conjugates on DPPH free radicals. (**B**) Surface hydrophobicity of CA-BLF non-covalent and covalent complexes. (**C**) Solubility of CA-BLF non-covalent and covalent complexes. (**D**) Calorimetric analysis of BLF, CA-BLF non-covalent complexes, and CA-BLF covalent conjugates. (**E1**) Foaming properties of CA-BLF. (**E2**) Emulsification properties of CA-BLF. (**F**) Digestion of CA-BLF was simulated in vitro. Different lowercase letters in the figure indicate significant differences (*p* < 0.05). Different lowercase letters in the figure indicate significant differences (*p* < 0.05). Uppercase letters represent differences between groups, while lowercase letters represent differences within groups.

As shown in Figure 7A, the scavenging activity of BLF on DPPH free radicals measured by this method was about 2.5%, only 1.2% lower than the antioxidant activity of the CA-BLF physical complex, indicating that the polyphenol–protein non-covalent complex can improve the antioxidant activity of proteins. The antioxidant properties of CA-BLF covalent compounds reached more than 20%, indicating that polyphenols and protein hydrolysates exhibited synergistic antioxidant activities when conjugated compounds were formed, much higher than those of non-covalent compounds. Antioxidant activity increased as their total polyphenol content increased, possibly because polyphenols introduced hydroxyl groups into the protein. This was similar to the results of Jiang et al., who found that when whey protein and casein were coupled with chlorogenic acid, free radical scavenging capacity increased significantly in a dose-dependent manner. Furthermore, there was no notable disparity in the total phenol content between the radical-grafted and physical complexes. However, the radical conjugate exhibited greater antioxidant activity than the physical complex, which might be attributed to the synergistic effect of CA and BLF in free radical conjugates [45]. In summary, CA-BLF conjugates can be used to enhance the antioxidant properties of BLF to improve the oxidative stability of several foods.

### 3.10. Surface Hydrophobicity (S_0_)

S_0_ affects protein conformation and emulsification [46]. Figure 7B shows the S_0_ values for BLF, non-covalent, and covalent complexes. Due to the introduction of polar groups of phenolic compounds and the exposure of some buried hydrophilic regions, the fluorescence intensity of BLF and CA was enhanced, and the surface hydrophobicity was reduced. The surface hydrophobicity of Rad-CA is the strongest, while that of Lac-CA is the most hydrophobic. This may be because laccase-catalyzed products have fewer effective binding sites for fluorescent probes. Differences in surface hydrophobicity between protein molecules may be due to the introduction of a large number of hydroxyl groups in the aromatic ring.

### 3.11. Solubility

The solubility of a protein is influenced by various factors, which is a crucial characteristic that affects its function. According to Figure 7C, the solubility of the protein increases after binding with polyphenols. The alteration in binding potential indicates that the interaction between protein molecules and phenolic compounds may modify the net charge and, therefore, affect the solubility behavior of the derivatives [47]. In comparison to non-covalent complexes, covalent complexes have higher solubility due to the binding of more CA, resulting in an increased content of phenolic hydroxyl groups in the system. Additionally, polyphenol binding reduces the surface hydrophobicity of the protein, thereby increasing its solubility. It is worth noting that Lac-CA has the weakest surface hydrophobicity and solubility. This is because the concentration of polyphenols is higher than that of protein binding sites. Under the catalysis of laccase, proteins and polyphenols may form clumps, leading to reduced protein solubility [18]. This is corroborated by the previous electrophoretic strip results. 

### 3.12. Measurement of Thermal Stability 

Protein–polyphenol interactions may lead to changes in the thermal stability of couplers, possibly resulting in enthalpy changes [11]. Therefore, the thermal stability of BLF and CA-BLF couplings was determined by DSC analysis (Figure 7D). The DSC spectra of BLF showed that it underwent a single endothermic transition with a peak temperature of about 71.95 °C. There was also a single endothermic transition in the DSC spectra of CA-BLF non-covalent and covalent complexes below 120 °C. The endothermic transition temperatures of alkali-treated CA-BLF covalent and non-covalent complexes increased. At the same time, those of other groups decreased, indicating that the non-covalent interaction of CA-BLF and alkali-treated CA-BLF would increase the denaturation temperature of BLF and improve the thermal stability of BLF. Ojha et al. showed that ferulic acid interacts with natural bovine serum albumin under neutral conditions and its melting temperature increases. This indicated that ferulic acid binding improved the thermal stability of bovine serum albumin [48]. In addition, the enthalpy change amplitude in the CA-BLF covalent complex treated by radical grafting and alkali was significantly smaller than that of BLF, indicating that the protein structure after polyphenol attachment was significantly changed compared with that treated by laccase [49].

### 3.13. Foaming Properties and Emulsifying Properties

Compared to BLF without polyphenols, BLF with polyphenols exhibited a significant increase in foam expansion rate and stability (*p* < 0.05). The improved foaming properties of protein–polyphenol complexes and splices may be attributed to larger shear deformation during agitation or better water solubility, which increases the adsorption rate of protein molecules at the air–water interface [50]. Figure 7E1 demonstrates that the interaction with CA enhances the foaming properties and foam stability of BLF. Rad-CA also exhibits good foaming properties and stability due to its high solubility.

The emulsification activity index (*EAI*) and emulsification stability index (*ESI*) of BLF and CA-BLF complexes are shown in Figure 7E2. Compared with BLF, adding CA significantly increased *EAI* (*p* < 0.05). The *ESI* of CA-BLF covalent complexes was higher than that of BLF except for covalent complexes (*p* < 0.05). This may be because covalent binding exposes more aromatic residues after polyphenol interactions, resulting in a higher affinity for the oil–water interface for proteins [51]. These results indicate that both covalent and non-covalent modifications may affect the emulsification performance of BLF. Among the three covalent binding modes, Lac-CA showed higher *EAI* and *ESI*. This may be because the laccase-catalyzed complex reduces the interfacial tension between the oil and water phases, promoting the formation of stable small droplets of emulsion.

### 3.14. Biological Accessibility

Lactoferrin is easily digested early in the stomach, which affects its function when it reaches the intestine. Therefore, it is important to understand the effect of different binding modes of CA on the gastrointestinal stability of BLF. After the addition of CA, the retention rate of BLF in the intestine was higher than that in the stomach, as shown in Figure 7F. The improvements in Rad-CA and Lac-CA were the best. This may be because the covalent interaction is irreversible, making the structure of BLF more stable and making it difficult for pepsin to exert its activity at the active site of BLF. According to the above results, the combination of CA and BLF can promote the absorption and utilization of BLF.

After binding with CA, the functional activity of BLF was improved, and the covalent effect was stronger than the non-covalent effect. The best way for CA to combine with BLF is alkaline, which can produce more stable covalent binding, protect it from the effect of stomach acid on BLF decomposition, and improve the absorption and utilization of BLF by the human body. Therefore, this study provides valuable insights into the design of lactoferrin-based colloidal delivery methods to encapsulate and protect bioactive ingredients, which can help in the design efficient delivery systems. It is worth noting that whether CA-BLF binding itself is toxic, and whether its biological properties are better than those of the pure BLFs tested, remains to be further discussed and studied.

## 4. Conclusions

The effects of non-covalent and covalent binding of lactoferrin (BLF) and chlorogenic acid (CA) on protein structure and function were studied. Conjugates were synthesized by laccase-catalyzed oxidation, free radical grafting, and alkali treatment. The covalent binding between BLF and CA was confirmed by sodium dodecyl sulfate–polyacrylamide gel electrophoresis (SDS-PAGE). Multispectral experiments characterized the BLF-CA complexes and their conjugates. The results showed that the tertiary structure of the protein in the BLF-CA covalent conjugate was changed. Combining the results of molecular docking and molecular dynamics, it was found that hydrogen bonds and electrostatic forces are the main non-covalent forces between CA and BLF. 

## Figures and Tables

**Table 1:** Grafting efficiency of CA-BLF physical complexes and CA-BLF conjugates.

Samples	Grafting Efficiency (%)
Physical complexes	12.63 ± 1.58 ^c^
Laccase catalytic oxidation	51.39 ± 0.28 ^b^
Free radical grafting	16.82 ± 4.62 ^c^
Alkali treatment	82.40 ± 2.53 ^a^

Different lowercase letters indicate significant differences between samples in the same column (*p* < 0.05).

**Table 2:** Stern–Volmer quenching constants, binding constants, number of binding sites, and thermodynamic parameters of lactoferrin–chlorogenic acid system at different temperatures.

T (K)	*K_sv_* (×10^4^ L mol^−1^)	*K_q_* (×10^12^ mol^−1^ s^−1^)	*K_A_* (×10^3^ L mol^−1^)	n	ΔH (kJ mol^−1^)	ΔS (J mol^−1^ K^−1^)	ΔG (kJ mol^−1^)
290	7.11	7.11	5.29	1.19	−1.35	85.79	−26.23
300	7.21	7.21	5.19	1.20			−27.09
310	7.44	7.44	5.10	1.22			−27.94

## Data Availability

The original contributions presented in the study are included in the article, further inquiries can be directed to the corresponding author.

## Abbreviations

BLF: bovine lactoferrin; CA: chlorogenic acid; SDS-PAGE: sodium dodecyl sulfate–polyacrylamide gel electrophoresis; CD: circular dichroism; FTIR: Fourier transform infrared spectroscopy; DPPH: 1,1-diphenyl-2-trinitro phenylhydrazine; DSC: differential scanning calorimetry.
